# Error-Driven Retrieval in Agreement Attraction Rarely Leads to Misinterpretation

**DOI:** 10.3389/fpsyg.2019.01002

**Published:** 2019-05-07

**Authors:** Zoe Schlueter, Dan Parker, Ellen Lau

**Affiliations:** ^1^Department of Linguistics, University of Maryland, College Park, MD, United States; ^2^Department of Linguistics and English Language, The University of Edinburgh, Edinburgh, United Kingdom; ^3^Linguistics Program, Department of English, College of William & Mary, Williamsburg, VA, United States

**Keywords:** sentence processing, comprehension, grammatical agreement, memory retrieval, similarity-based interference, agreement attraction

## Abstract

Previous work on agreement computation in sentence comprehension motivates a model in which the parser predicts the verb’s number and engages in retrieval of the agreement controller only when it detects a mismatch between the prediction and the bottom-up input. It is the error-driven second stage of this process that is prone to similarity-based interference and can result in the illusory licensing of a subject–verb number agreement violation in the presence of a structurally irrelevant noun matching the number marking on the verb (‘*The bed by the lamps were…*’), giving rise to an effect known as ‘agreement attraction’. Here we ask to what extent the error-driven retrieval process underlying the illusory licensing alters the structural and thematic representation of the sentence. We use a novel dual-task paradigm that combines self-paced reading with a speeded forced choice task to investigate whether agreement attraction leads comprehenders to erroneously interpret the attractor as the thematic subject, which would indicate structural reanalysis. Participants read sentence fragments (‘*The bed by the lamp/lamps was/were undoubtedly quite*’) and completed the sentences by choosing between two adjectives (‘*comfortable*’/’*bright*’) which were either compatible with the subject’s head noun or with the attractor. We found the expected agreement attraction profile in the self-paced reading data but the interpretive error occurs on only a small subset of attraction trials, suggesting that in agreement attraction agreement checking rarely matches the thematic relation. We propose that illusory licensing of an agreement violation often reflects a low-level rechecking process that is only concerned with number and does not have an impact on the structural representation of the sentence. Interestingly, this suggests that error-driven repair processes can result in a globally inconsistent final sentence representation with a persistent mismatch between the subject and the verb.

## Introduction

Much recent work has asked whether the interpretation comprehenders arrive at always tracks the syntax. We pursue this issue by investigating whether the illusory licensing of an agreement violation (‘*The key to the cabinets are rusty*’), known as agreement attraction, reflects a change in the structural and thematic representation of the sentence or a low-level rechecking operation. Previous work has shown that when comprehenders receive input that cannot be integrated into the current parse, they often engage in structural reanalysis of the previous input. This illustrates that an error signal can cause restructuring, but does a grammatical illusion like agreement attraction also reflect structural reanalysis? If the error signal from an agreement violation triggers similar reanalysis, the structural representation would be consistent with the grammar and the attractor would be misinterpreted as the subject. Although the interpretation would differ from the input, it would be consistent with the structure of the mental representation. However, if agreement attraction is the result of a simple rechecking operation the final representation contains an agreement violation. Here, we show that the illusory licensing of subject–verb number agreement generally does not lead to the misinterpretation of the attractor as the thematic subject, suggesting that most instances of agreement attraction do not reflect a structural reanalysis when the attractor is misretrieved in the search for the agreement controller in memory. Instead, we propose that error-driven retrieval of the agreement controller generally involves a low-level number rechecking operation.

### Structure and Interpretation

In the past 15 years there has been mounting evidence that the interpretations comprehenders arrive at are not always uniformly consistent with the linguistic input (for recent reviews see Ferreira, 2003; Christianson, 2016; Karimi and Ferreira, 2016). Renewed interest in this question was first sparked by work by Ferreira and colleagues, who showed that after reading garden-path sentences like ‘*While Anna dressed the baby played in the crib*’, participants would frequently accept interpretations not consistent with the input, answering ‘yes’ when asked if Anna had dressed the baby (Christianson et al., 2001; Ferreira et al., 2001). Ferreira and colleagues initially considered an ‘erroneous structure’ view, concluding that comprehenders do not always recover completely from the initial misparse in garden-path sentences. However, more recent research (Slattery et al., 2013) suggests that the lingering misinterpretation observed with garden-path sentences is not a result of the parser’s failure to completely reanalyze the structural representation, but a failure to suppress the initial interpretation. In other words, if both parsing and interpretation are incremental, then the initial (erroneous parse) will have been interpreted even if the syntactic parse is successfully reanalyzed at the point of disambiguation. Therefore, the interpretation of the initial misparse is not licensed by the final input, but it is consistent with an interpretation derived from the structure during processing. Slattery et al. (2013) argued that this interpretation lingers in memory and can impact end-of-sentence judgments, even if the ultimate syntactic parse – and the ultimate sentence-level interpretation – is consistent with the input.

Misinterpretations have recently also been observed for implausible but syntactically unambiguous sentences. Gibson et al. (2013) found that participants frequently answered comprehension questions about implausible sentences (like ‘*The mother gave the candle the daughter*’) not based on the grammatically licensed interpretation, but rather on a plausible alternative (here ‘*The mother gave the candle to the daughter*’). Gibson et al. (2013) argued that such effects can be explained by a noisy channel model of language comprehension (e.g., Levy, 2008; Levy et al., 2009). Interestingly, there is evidence that comprehenders not only generate a plausible interpretation that is not licensed by the linguistic input, but that they actually build a syntactic representation of the unlicensed interpretation. For instance, implausible sentences with a double object construction have been found to syntactically prime the prepositional dative construction of the plausible alternative (Slevc and Momma, 2015). This finding is consistent with a speech error reversal system proposed by Frazier and Clifton (2015); Frazier (2015). According to this account, comprehenders use their knowledge of the production system – specifically, what kind of speech errors frequently occur – to repair the input they receive. Similar proposals have also been made to account for the systematic misinterpretation of antecedent-ellipsis mismatches (Arregui et al., 2006; Frazier, 2013; but cf. Parker, 2018).

Misinterpretations are not random and arise systematically: garden-path sentences, implausible sentences, and other types of mismatches present instances in which the interpretation is not licensed by the actual linguistic input, but is licensed by the structure that is assigned to the input at some stage during processing. In these cases, the parser engages in structural reanalysis when it encounters an error signal from the bottom-up input. For instance, in the case of garden-path sentences, the misinterpretation arises before the parser engages in reanalysis of the input and then lingers, whereas for implausible sentences the error signal is semantic in nature (the comprehender arrives at an interpretation that they believe was not the intended speaker meaning) and leads to reanalysis that is not consistent with the actual input. Importantly for us, this suggests that the parser frequently engages in structural reanalysis in response to error signals and that misinterpretations are systematically linked to structures assigned to the input which are consistent with the misinterpretation.

In summary, there is clear evidence that under certain circumstances comprehenders systematically generate interpretations that are not faithful to the linguistic input. However, it seems possible that this involves building grammatically well-formed structural representations that are consistent with the misinterpretation, though not completely faithful to the input. Here, we ask whether misretrieval due to similarity-based interference in subject–verb agreement attraction is another source of systematic misinterpretation. In the following sections we outline the mechanisms underlying agreement attraction and how they might interact with interpretation.

### Subject–Verb Agreement Attraction

Subject–verb agreement in English is a morphosyntactic dependency in which the number feature on the verb has to match the number feature of the subject. This dependency is susceptible to so-called “agreement attraction” errors, in which the number marking on the verb matches a structurally inaccessible plural noun rather than the singular subject (‘*The key to the cabinets are rusty*’). Agreement attraction occurs not only in production (Bock and Miller, 1991), but also in comprehension, where these sentences are often perceived as grammatical and do not show the processing cost normally associated with agreement violations (e.g., Pearlmutter et al., 1999; Wagers et al., 2009). This facilitation can be accounted for by a memory architecture based on cue-based retrieval (Wagers et al., 2009; Dillon et al., 2013; Tanner et al., 2014; Lago et al., 2015; Tucker et al., 2015). Sentence processing frequently requires comprehenders to establish dependencies between items that are not directly adjacent to each other, which means that retrieving items from memory is central to language comprehension. According to cue-based retrieval models (e.g., McElree, 2000; Van Dyke and Lewis, 2003; Lewis and Vasishth, 2005), items are encoded in memory as bundles of features and are content-addressable based on the features they contain (Lewis et al., 2006). When retrieval is triggered, the retrieval cues available at the retrieval site are used to access the target item in memory. Activation from each cue is transferred to each item with a matching feature and the item with the highest activation level is retrieved. When the target is a perfect match for all the retrieval cues, a partial match between the cues and a non-target item will not prevent it from being retrieved. However, when there is a partial mismatch between the target’s features and the cues, the presence of a partially matching non-target item can lead to the misretrieval of this non-target item, in what is known as “similarity-based interference”.

In the case of subject–verb agreement, the retrieval cues on the verb include both structural and number cues, e.g., [+subject] and [+plural] (see Arnett and Wagers, 2017, for discussion of the subject cue). When there is a number mismatch between the subject and the verb in the presence of a plural non-subject attractor (i.e., ungrammatical sentences like, ‘*The key to the cabinets are…*’), the activation from the number cue raises the level of activation of the attractor, but not the subject. In a subset of cases, this leads to the misretrieval of the number-matching attractor instead of the number-mismatching subject. This is reflected in higher acceptance rates and an amelioration of the processing difficulty associated with agreement violations in online measures.

In a cue-based retrieval model of agreement attraction there are two theoretical possibilities about when retrieval of the agreement controller is triggered. In principle, it is possible that subject–verb agreement processing in comprehension always involves retrieval of the agreement controller from memory, regardless of whether the verb and subject match in number. In grammatical sentences, the subject’s features are a perfect match for the retrieval cues on the verb: it fulfills both the structural cue of being the subject and its number feature matches the number cue. Even if there is a structurally irrelevant noun that matches the number marking on the verb, this item only receives activation from one of the retrieval cues. Its activation level is therefore lower than that of the subject (modulo effects of noise). Consequently, the appropriate target is retrieved from memory. Retrieval in a sentence with an agreement violation would be triggered in the same way (by default), but the outcome would be different.

The second possibility under a cue-based retrieval account is that the retrieval-process underlying agreement attraction is an error-driven phenomenon (Wagers et al., 2009; Lago et al., 2015) that occurs only when the verb and subject mismatch in number (i.e., ungrammatical sentences). There is overwhelming evidence that language comprehension is not exclusively driven by bottom-up input and that comprehenders deploy top-down mechanisms to make use of existing information to predict upcoming input (see Kutas et al., 2011, for review). In the case of subject–verb agreement, this motivates a view in which comprehenders predict the number of the upcoming verb based on the number feature of the subject. If the bottom-up input matches their prediction, the verb’s number marking is licensed and there is no need to retrieve the agreement controller. However, when the prediction is violated, this triggers error-driven retrieval of the agreement controller. Under this model, grammatical sentences without an agreement violation do not involve cue-based retrieval. Instead, agreement checking is a two-stage process and the second step (retrieval) is limited to instances where an agreement violation has been detected.

An important type of evidence in favor of this two-stage model are data suggesting that comprehenders initially show sensitivity to the agreement violation even in the presence of a number-matching attractor. Recent research has shown that attraction effects occur in the right tail of the reading time distribution, compared to the effect of grammaticality which also exerts an influence on faster reading times (Staub, 2009, 2010; Lago et al., 2015). Moreover, in eye-tracking studies, agreement violations have been observed in early reading time measures, while attraction effects were only found in late reading time measures (Dillon et al., 2013; Parker and Phillips, 2017). This suggests that during the initial processing of the verb comprehenders are sensitive to the agreement violation even in the presence of a plural attractor. The amelioration of the processing disruption associated with this violation does not occur until a later stage of processing.

### Agreement and Interpretation

While this study focuses on the question whether agreement attraction leads to the misinterpretation of the local noun as the thematic subject, it should be noted that a separate question relating to agreement and interpretation is whether attraction cases reflect instances where the number of the subject is misrepresented as plural. Representational models relying on feature percolation or spreading activation like those often assumed for agreement attraction in production (e.g., Bock and Eberhard, 1993; Pearlmutter et al., 1999; Bock et al., 2004; Eberhard et al., 2005) have sometimes been proposed to extend to comprehension (Pearlmutter et al., 1999). The question whether comprehenders mistakenly interpret the subject as plural is central to representational accounts of agreement attraction in comprehension but has only rarely been directly addressed in previous studies.

One study that did investigate the subject’s number representation in agreement attraction was conducted by Patson and Husband (2016). This study used self-paced reading followed by comprehension questions that explicitly probed participants’ interpretation of the subject’s number feature: a sentence like *‘The key to the cabinets are on the table*’ was followed by the question ‘*Was there more than one key?*’. Comprehenders were more likely to agree that there were multiples of the entity denoted by the singular head noun when there was a plural attractor or a plural verb. This effect was strongest in agreement attraction configurations, in which both the attractor and the verb were plural. This study was recently replicated and extended by Brehm et al. (2019), who observed the same pattern of results to the comprehension questions, and additionally found that non-literal interpretations were more likely when the sentence was assumed to be produced by a native speaker of standard American English compared to an L2 speaker or a speaker of a regional dialect. Based on these studies, it does seem that comprehenders do indeed sometimes misrepresent the number of the complex subject noun phrase.

However, for both Patson and Husband (2016) and Brehm et al. (2019), non-literal answers about the number of the subject occurred not only in agreement attraction configurations, but whenever there was a plural feature present on the attractor or the verb. While a non-literal answer in the presence of a plural attractor would support a representational account of agreement attraction in comprehension, there are two reasons why the data overall suggest a somewhat different explanation. First, non-literal answers were also more common when the local noun was singular and only the verb was plural, which is not predicted under representational accounts of agreement attraction. As Brehm et al. (2019) point out, this is consistent with a noisy channel model of comprehension, in which comprehenders make rational inferences about the intended meaning of anomalous utterances. Second, Patson and Husband’s self-paced reading data are not consistent with the automatic misrepresentation of complex noun phrases, as it shows no evidence of disrupted processing at the verb in grammatical sentences when the attractor was plural (‘*The key to the cabinets was…*’). If comprehenders misrepresent the number feature of the subject in the presence of a plural attractor, this should be reflected in processing difficulties at the verb in grammatical sentences with plural attractors. One alternative explanation of the comprehension results in these studies is that answers to explicit comprehension questions are not always an accurate reflection of the representation built during the earlier processing of the sentence.

In fact, a recent series of experiments by Dempsey et al. (2016) and Tanner et al. (2018) is consistent with this alternative explanation. In a self-paced reading task, they used items in which a complex noun phrase with a singular head noun and either a singular or plural noun inside a prepositional modifier was introduced as the object in the first sentence and then referred back to by a singular or plural noun phrase as the subject of the second sentence [‘*My husband placed the newspaper with the perfume ad(s) on the kitchen table. The newspaper(s) looked muddy …*’]. They did not find any facilitation in the processing of a co-referential plural noun phrase when the noun inside the prepositional modifier was plural. This indicates that the complex NP’s number information had not been misrepresented as plural by virtue of containing a plural element. In spite of this, a quasi-replication of Patson and Husband’s study with the same materials as the self-paced reading task showed that follow-up comprehension questions about the number of the entity denoted by the complex NP were affected by the presence of a plural noun inside the prepositional modifier. Tanner et al. (2018) argued that, when taken together with the self-paced reading data, this shows that comprehension question accuracy might not directly reflect the misrepresentation of NP number during processing. Instead, they proposed a feature misbinding account according to which direct metalinguistic questions might lead to the retrieval of “floating” plural features that are not bound to their lexical hosts in memory. In the Discussion, we return to the question of number misinterpretation and whether agreement attraction in comprehension might result in, rather than stem from, misrepresenting the subject as plural.

Although representational models can account for the agreement attraction data in production, they fail to capture some of the comprehension data. If agreement attraction is a result of misrepresenting the number feature of the subject, this predicts that grammatical sentences should sometimes be perceived as ungrammatical in the presence of a plural attractor (‘*The key to the cabinets is*…’). However, that does not seem to be the case (Wagers et al., 2009; Lago et al., 2015; Tucker et al., 2015; but cf. Pearlmutter et al., 1999). Cue-based memory retrieval models provide a good account of the formation of morphosyntactic dependencies such as subject–verb agreement in sentence processing. However, the ultimate goal of comprehension is not to establish dependencies between items to check formal features, but to derive the intended interpretation by building a structural representation of the input. We therefore ask if the output of memory retrieval operations for checking formal features changes the structural representation and interpretation of a sentence.

Under a two-step model of agreement attraction, encountering an agreement violation is an error signal from the bottom-up input. As previously discussed, the parser frequently engages in structural reanalysis when it encounters error signals, for example at the point of disambiguation in garden-path sentences. However, it should be noted that the proposed reanalysis in agreement attraction would be fundamentally different from reanalysis in garden-path sentences. In a garden-path sentence, it is simply impossible to integrate the new input into the existing structure without violating structural constraints. In contrast, when the parser encounters a subject–verb agreement violation, the structural configuration for integrating the verb is there. There is only a mismatch between one of the predicted features (number) and the bottom-up input. If reanalysis is costly, it might only be deployed when the error-signal is triggered by a severe violation. Moreover, in garden path sentences, the parser assigns a different analysis to the entire previous input. In agreement attraction, misrepresenting the attractor as the subject would require excluding some of the previous input from the newly built structure. In a sentence like ‘*The key to the cabinets are old*,’ if the attractor (‘*the cabinets*’) is misanalyzed as the subject due to misretrieval in agreement checking, there is no clear way for the subject’s actual head noun to be incorporated into this revised structure. Reanalysis might only be possible if the input that has already been assigned a structure can be completely integrated into the new structure.

If agreement attraction involves reanalysis and the retrieval output is integrated in the subject position, this would lead to misinterpretation of the attractor as the thematic subject. The interpretation would not be consistent with the linguistic input, but not because comprehenders are engaging in shallow parsing. Instead, the misinterpretation would be a systematic result of the basic properties of the memory system subserving language comprehension. Here, we briefly review the studies that we are aware of that address the question of whether the attractor is misanalyzed as the subject in agreement attraction.

Thornton and MacDonald (2003) conducted a series of experiments examining the impact of whether the attractor was also a plausible subject for the verb. In two production studies, participants were presented with a preamble containing two nouns (‘*The album by the classical composers*’) and a verb that had to be used to form a complete sentence. They manipulated whether the verb could have both the head noun and the attractor or only the head noun as a plausible (passive) subject and found that agreement attraction error rates were increased when the plural attractor was a plausible subject. The comprehension experiment also showed plausibility effects as reflected in an increase in reading time at the verb in the presence of a plural attractor when both the head noun and the attractor were a plausible subject, which is reminiscent of the semantic interference found by Van Dyke and McElree (2006). However, the comprehension experiment did not include ungrammatical sentences to test for agreement attraction effects. Therefore, the data is not directly informative about how misretrieval for formal feature checking can alter interpretations in comprehension.

Pittman and Smyth (2005) replicated Thornton and MacDonald’s production results and added a new component to the elicited production task in order to investigate whether participants had misrepresented the attractor as the subject in cases where they produced agreement errors. After repeating the preamble and completing the sentence using the given predicate, which was either plausible with both the head noun and the local noun or only with the head noun, participants were presented with a choice of two predicates. They had to continue the sentence using ‘*and*’ followed by whichever of the two predicates they chose. One of the predicates was always a semantic match for the head noun and the other for the attractor. For example, for a preamble like ‘*The boy by the trees*’ with the first predicate ‘*tall*’ (matching both head and attractor) or ‘*playful*’ (matching only the head), the choice would be between ‘*chubby*’ and ‘*green*.’ As in previous studies on agreement attraction in production, preambles with a singular head noun and a plural local noun led to the production of more agreement errors. The agreement error rate was higher when the local noun was a plausible subject of the first predicate and the selection error rate for the second predicate was higher in trials in which participants had produced an agreement error. According to Pittman and Smyth, this shows that participants sometimes got confused about which of the nouns was the thematic subject during the planning stage of production and a subset of the agreement errors were a reflection of this confusion. While this suggests that in an elicited production task the attractor might sometimes be misinterpreted as the thematic subject, these data do not allow us to draw conclusions about the impact of misretrieval on the structural representation of the sentence in comprehension. Not only are agreement attraction in production and comprehension often attributed to different mechanisms (Acuña-Fariña, 2009, 2012; Acuña-Fariña et al., 2014; Tanner et al., 2014), but the misinterpretation in this case arose during the message planning stage, which does not apply to comprehension. However, as outlined above, if the retrieval output for agreement checking is used to change the existing parse of the sentence, a possible consequence of misretrieval in agreement attraction is that comprehenders might misinterpret the attractor as the subject of the sentence.

Lau et al. (2008) used inverted pseudoclefts in a self-paced reading experiment to address the question whether the attractor is misinterpreted as the subject by testing for plausibility effects at the thematic verb. They used sentences like ‘*The phone by the toilets was/were what Patrick used/dialed/flushed/embarrassed*,’ in which they manipulated grammaticality as well as the plausibility of the head noun and the attractor as thematic subjects by varying the verb. If agreement attraction triggers structural reanalysis and the misrepresentation of the attractor as the thematic subject, the plausibility match between the attractor and the verb should matter. However, the results only show a main effect of head noun plausibility with participants exhibiting a slow-down at the thematic verb when the head noun of the subject was not a plausible match. There was no interaction with attraction context or the plausibility of the attractor. Lau et al. (2008) conclude that the misretrieval of the attractor does not lead to thematic subject reassignment, meaning that the misretrieval is selective for formal feature satisfaction. However, this study used inverted pseudoclefts, which is not a structure used in other agreement attraction studies. It requires retrieval of the subject not just for agreement checking at the inflected auxiliary, but again at the wh-word before the main verb is encountered, which might have influenced their results. We address this question by using a dual-task design that provides a very clear measure of which noun phrase comprehenders took to be the subject.

## The Present Study

We used a novel dual-task paradigm to investigate whether agreement attraction leads comprehenders to erroneously interpret the attractor as the subject of the sentence. Misinterpretation of the attractor as the thematic subject would indicate that the retrieval output for agreement checking is used to alter the structural representation of the sentence. We developed a dual-task paradigm combining self-paced reading with a forced-choice task. Participants read sentence fragments and had to complete them by selecting an adjective that was either compatible with the head noun of the subject or the attractor noun. The choice of adjective on each trial is indicative of whether the attractor was misrepresented as the subject. If erroneously retrieving the attractor in the process of agreement checking leads to the reanalysis of the attractor as the subject, we expect to see a higher rate of participants choosing the adjective that matches only the attractor in an agreement attraction configuration, i.e., with an ungrammatical verb and a plural attractor. If, however, the error-driven retrieval process in agreement checking is has no impact on the structural representation, comprehenders should not be more likely to choose the attractor-matching adjective in the agreement attraction condition.

The nature of the dual-task paradigm also makes it possible to analyze not only adjective choice and overall reading times, but to take adjective choice on each trial into consideration when analyzing reading times. Overall, we expected to find a typical agreement attraction profile for the self-paced reading data, i.e., a slow-down in ungrammatical conditions, ameliorated by the presence of a plural attractor. If agreement attraction causes comprehenders to mistake the attractor for the subject, this should be reflected by choosing the attractor-matching adjective. Consequently, in the reading time data we would expect an attraction effect for trials on which the attractor-matching adjective was chosen. In contrast, we would expect to see less attraction for trials that culminated in a head-matching adjective choice. However, if misretrieval of the attractor does not result in reanalysis, the reading time data should show agreement attraction regardless of adjective choice.

### Participants

Sixty-four native speakers of American English were recruited via Amazon Mechanical Turk for monetary compensation. All participants in this experiment and both norming studies described below provided informed consent and underwent a screening for native speaker abilities. This screening probed knowledge of the constraints on English morphology, tense, modality, ellipsis, and syntactic islands.

### Materials

There were 48 items sets in 4 conditions. Each item consisted of a sentence fragment for self-paced reading and two adjectives for the sentence-final adjective-choice task. The sentence fragments all had a complex subject with a singular head noun and a prepositional modifier containing the attractor. The subject was followed by an inflected form of ‘*be*’ and two adverbs. The sentence-final adjective was displayed as a forced-choice task: one adjective was a plausible match only for the head noun of the subject and the other only for the attractor, as illustrated in (1). We manipulated attractor number (singular/plural) and grammaticality (grammatical/ungrammatical). The full set of experimental items can be found in the Supplementary Materials.

(1)
  (a) The boy by the tree is really very CHUBBY/GREEN
  (b) The boy by the tree are really very CHUBBY/GREEN
  (c) The boy by the trees is really very CHUBBY/GREEN
  (d) The boy by the trees are really very CHUBBY/GREEN

The items were distributed across four lists in a Latin Square design. In addition to the 48 experimental items, each list also contained 72 filler items of similar syntactic complexity for which participants also had to choose between two possible sentence-final completions.

#### Plausibility Norming

Since the premise of the dual-task paradigm is that the adjective choice is informative about whether the participant has misinterpreted the attractor as the thematic subject, it is crucial that one of the adjectives is semantically plausible only for the head noun and the other only for the attractor. We conducted a plausibility rating study of simple sentences with potential head nouns and attractor nouns in subject position, varying the predicative adjective. The aim was to select 48 item sets in which one of the adjectives was rated highly plausible only for the head noun and the other only for the attractor.

Thirty native speakers of English participated in an adjective norming study on Ibex in which they rated 66 items in 6 conditions for plausibility on a scale from 1 (very implausible) to 7 (very plausible). These participants did not participate in the other norming study or the main experiment. All items were grammatical and the task also included 18 plausible fillers, 16 implausible fillers and 7 control items. We constructed 66 preliminary items containing a complex subject with a prepositional modifier, followed by an inflected form of *be*, two adverbs, and a sentence-final adjective. For each item, there were 8 conditions, crossing attractor number, grammaticality, and adjective plausibility. Based on these preliminary items, we constructed 66 item sets for norming, manipulating whether the subject was the head noun or the attractor noun in the 66 preliminary items. Apart from subject type (head noun vs. attractor), we also manipulated adjective type (head-match vs. attractor-match), and subject number. Since in the materials for the dual-task paradigm the head noun of the subject is always singular, the norming study included plural versions only of the attractors. This led to a total of six conditions, as illustrated in (2). The ratings were used to calculate the average plausibility ratings for the plausible conditions (a, d, f) and the implausible conditions for each item (b, c, e). We then selected the 48 items with the greatest difference between plausibility ratings for the plausible and the implausible conditions.

(2)
  (a) The **boy** is really very *chubby*.
  (b) The **boy** is really very *green*.
  (c) The **tree** is really very *chubby*.
  (d) The **tree** is really very *green*.
  (e) The **trees** are really very *chubby*.
  (f) The **trees** are really very *green*.

#### Agreement Attraction Norming

The 48 chosen items were then used in a speeded acceptability judgment task to confirm that they caused the expected agreement attraction effect. 24 native speakers of American English read sentences presented word-by-word in the center of the screen with a stimulus onset asynchrony of 400 ms (inter-stimulus interval: 100 ms). None of these subjects participated in the other norming study or the main experiment. Following each sentence, participants had 2,000 ms to indicate whether the sentence had been acceptable. The instructions explicitly asked them to judge sentences based on whether they sounded like natural English. There were 72 fillers (half grammatical) in addition to the 48 experimental items. In order to avoid exposing participants to a large number of implausible sentences, the sentence-final adjective was always the one compatible with the head noun of the subject. In the dual-task paradigm, the attraction effect in self-paced reading is measured on the verb and its spillover regions, before participants are presented with the adjectives.

**FIGURE 1:**
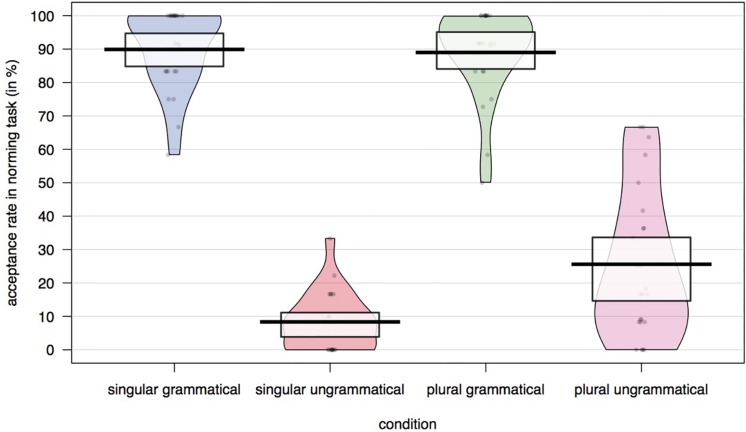
Acceptance rates across conditions in the speeded acceptability judgment task.

The acceptance rates across conditions were analyzed with a mixed-logit model (Jaeger, 2008), excluding trials on which no response was made within 2,000 ms (2.5% of all trials). The acceptance rates for each condition are plotted in Figure 1. Table 1 contains the results of the mixed-logit model with grammaticality and attractor model as fixed effects (sum-coded). The random effects structure included by-subject and by-item random intercepts and by-subject random slopes for grammaticality.^1^ As expected, grammatical sentences were more likely to be judged acceptable than ungrammatical sentences (89.4% vs. 16.7%). Sentences with a plural attractor were also more likely to be accepted than sentences with a singular attractor (49.5% vs. 57.1%), but this effect was driven by the higher rate of acceptance of ungrammatical sentences with plural attractors. Participants were more likely to accept an ungrammatical sentence when the number of the attractor was plural (25.1% for ungrammatical sentences with a plural attractor compared to 8.2% for those with a singular attractor). This indicates that comprehenders indeed experience attraction with this particular item set, making these materials suitable for the novel dual-task paradigm.

**TABLE 1:** Results of the mixed logit model in the speeded acceptability judgment task.

**Parameter**	**Estimate**	**Std. error**	***z*-value**	***p*-value**
Intercept	0.13	0.23	0.57	0.57
Grammaticality	2.41	0.17	13.89	<0.001
Attractor number	−0.39	0.10	−3.79	<0.001
Grammaticality × attractor number	0.45	0.10	4.31	<0.001

### Procedure

The sentences were presented in a self-paced reading paradigm with centered display using Ibex Farm (Drummond, 2019). Participants had to press the spacebar to see each new word and only one word at a time was visible. When they pressed the spacebar to reveal the final word of the sentence, the two adjectives for the forced-choice task appeared on the screen simultaneously, one to the left of the center and one to the right. The order in which the adjectives were displayed was randomized for each participant. Once the two adjectives appeared, participants had 3,000 ms to choose one of them by pressing the ‘f’-key for the one on the left or the ‘j’-key for the one on the right. If no response was made within 3,000 ms, the adjective-choice task timed out and the experiment moved on to the next trial.

### Analysis

Trials on which there was no response within the 3,000 ms time limit were excluded from all analyses reported here (1.4% of experimental trials, 42 of 3,072 trials). We analyzed responses to the adjective-choice task with a mixed logit model (Jaeger, 2008) using the lme4 package (Bates et al., 2015) in the R computing environment (R Core Team, 2018). The model included attractor number and grammaticality as fixed effects (sum-coded) and by-subject and by-item random intercepts. The model was initially fitted with the maximal random effects structure, which was then simplified until the model converged (Barr et al., 2013).

Although the main focus of the experiment was the adjective-choice task, we also analyzed the self-paced reading data. The regions of analysis were the verb and its spillover region (first adverb). Reading times of 0 ms and reading times exceeding a threshold of 2,000 ms were not included in the analysis, leading to the exclusion of less than 0.2% of experimental trials in each region of analysis.^2^ RTs were log transformed and analyzed using linear mixed effects models with attractor number, grammaticality and adjective choice as fixed effects. The final model included random by-subject and by-item intercepts. In addition, we also split the SPR data based on adjective choice on each trial and conducted a response-contingent RT analysis.

### Results

#### Adjective-Choice Task

**FIGURE 2:**
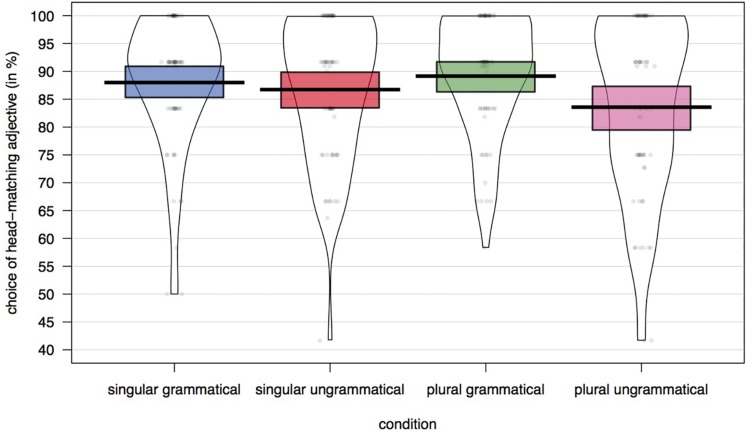
Percentage of trials with a head-matching adjective choice across conditions.

The percentage of trials on which a head-noun matching adjective was chosen for each of the experimental conditions is plotted in Figure 2 and the results from the model are presented in Table 2. There was a significant main effect of grammaticality (*p* < 0.01): participants were more likely to choose the adjective that matched only the subject’s head noun in grammatical than in ungrammatical sentences. There was also a significant interaction between grammaticality and attractor number (*p* = 0.03). In ungrammatical sentences participants were less likely to choose the head-matching adjective when the attractor was plural. As can be seen in Figure 2, the overall accuracy rate in the forced-choice task was very high. The rate of choosing the attractor-matching adjective was only 5.6% higher in the attraction condition (ungrammatical with a plural attractor: 16.6%) than in the grammatical condition with a plural attractor (10.8%), and only 3.2% higher than in the ungrammatical condition with a singular attractor (13.3%).

**TABLE 2:** Results of the mixed logit model for adjective choice.

**Parameter**	**Estimate**	**Std. error**	***z*-value**	***p*-value**
Intercept	2.57	0.23	11.39	<0.001
Grammaticality	0.18	0.06	3.00	<0.01
Attractor number	0.04	0.06	0.63	0.53
Grammaticality × attractor number	−0.13	0.06	2.16	0.03

**FIGURE 3:**
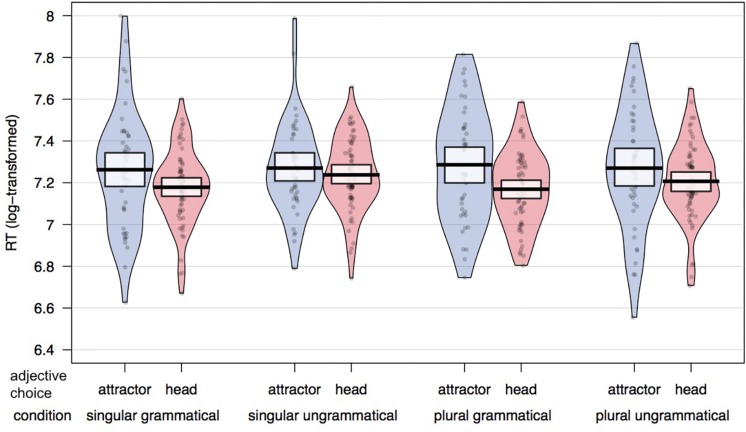
Mean RTs split by adjective choice (attractor-matching response in blue; head-matching response in red) in each experimental condition. Proportion of head noun compatible responses beneath condition labels.

Figure 3 plots raw RTs for head-matching and attractor-matching adjective responses across conditions. Results of the linear mixed effects model with fixed effects of grammaticality, attractor number and adjective choice are presented in Table 3. There was a significant effect of adjective choice (*t* = −3.17), with a slowdown in trials on which the attractor-matching adjective was chosen compared to when the head-matching adjective was chosen. The RT difference between head-compatible and attractor-compatible adjective responses was larger in the grammatical than the ungrammatical conditions. However, this interaction between grammaticality and adjective choice was only marginally significant (*t* = −1.95).

**TABLE 3:** Results of linear mixed effects model of response time on the adjective-choice task (using log transformed RTs).

**Parameter**	**Estimate**	**Std. error**	***t*-value**
Intercept	7.25	0.028	262.20
Grammaticality	<−0.01	0.012	−0.01
Attractor number	<−0.01	0.012	−0.14
Adjective choice	0.04	0.014	−3.17
Grammaticality × attractor number	−0.01	0.012	−0.85
Grammaticality × adjective choice	−0.03	0.013	−1.95
Attractor number × adjective choice	0.01	0.013	1.04
Grammaticality × attractor number × adj. choice	0.01	0.013	0.38

#### Self-Paced Reading

**FIGURE 4:**
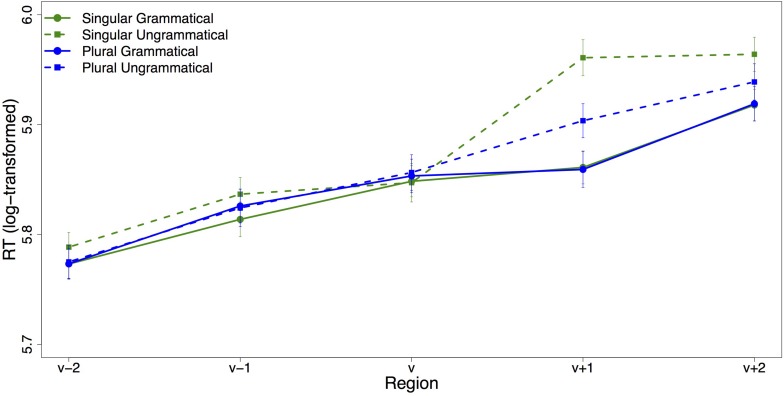
Region-by-region mean reading times. Error bars indicate standard error of the mean.

All analyses were performed on log transformed RTs. Table 4 contains the results of the linear mixed effects models for the verb region and the spillover region. The region-by-region average (log-transformed) reading times are plotted in Figure 4. The only significant effect in the verb region was a three-way interaction between grammaticality, attractor number and adjective choice (*t* = 2.48): Grammaticality had a larger effect on adjective-choice when the attractor was plural compared to when it was singular. In the spillover region, there was a main effect of grammaticality (*t* = −4.08), with increased reading times for ungrammatical sentences. There was also a main effect of attractor number (*t* = 2.02), with increased reading times for sentences with singular attractors, but the interaction between grammaticality and attractor number was not significant.

**TABLE 4:** Results of the linear mixed effects model (using log transformed RTs).

**Parameter**	**Estimate**	**Std. error**	***t*-value**
**Verb region**			
Intercept	5.849	0.043	136.49
Grammaticality	−0.003	0.008	−0.33
Attractor number	−0.004	0.008	−0.51
Adjective choice	−0.004	0.008	−0.51
Grammaticality × attractor number	0.014	0.008	1.91
Grammaticality × adjective choice	−0.004	0.008	−0.53
Attractor number × adjective choice	−0.002	0.008	−0.31
Grammaticality × attractor number × adjective choice	0.019	0.008	2.48
**Spillover region**			
Intercept	5.907	0.043	136.17
Grammaticality	−0.030	0.007	−4.08
Attractor number	0.015	0.007	2.02
Adjective choice	0.013	0.008	1.72
Grammaticality × attractor number	−0.008	0.007	−1.04
Grammaticality × adjective choice	0.006	0.008	0.75
Attractor number × adjective choice	<−0.001	0.008	−0.02
Grammaticality × attractor number × adjective choice	0.009	0.007	1.22

#### Response-Contingent Self-Paced Reading

**FIGURE 5:**
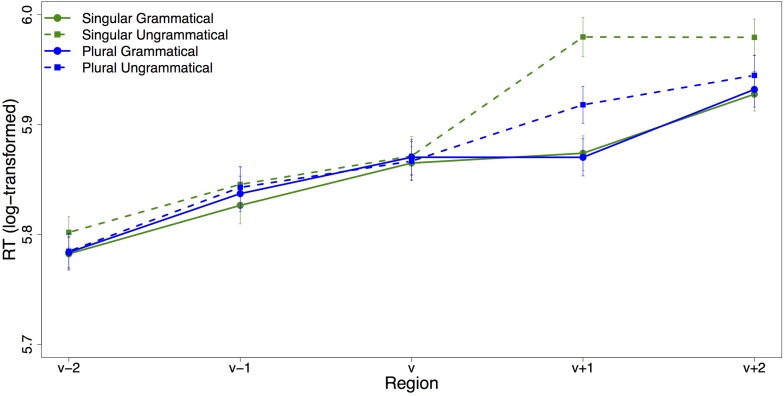
Region-by-region mean reading times for trials on which the (correct) head-matching adjective was chosen. Error bars indicate standard error of the mean.

The nature of the dual-task paradigm allows us to examine reaction time profiles of trials based on adjective choice. Figure 5 shows the average log-transformed reading time per region for each condition for trials on which the (correct) head-matching adjective was chosen. The plot looks almost identical to the overall SPR plot. Visually, there is a very clear slow-down for the ungrammatical conditions in the verb’s spillover region, which is ameliorated for ungrammatical sentences with a plural attractor. Statistical analysis confirms this: While there is no significant effect in the verb region, in the verb’s spillover region grammaticality, attractor number and their interaction had a significant effect on reading times (see Table 5). As expected, agreement violations led to a slowdown in the verb’s spillover region compared to sentences with correct subject–verb agreement, as reflected in the main effect of grammaticality (*t* = −6.67). Reading times in the spillover region were longer for sentences with a singular than a plural attractor (*t* = 2.78). This result was not expected and seems to be attributable to the large slowdown in the ungrammatical condition with a singular attractor: the large slow-down in the ungrammatical singular condition means that the average RT of the two singular conditions is significantly slower than the average RT of the two plural conditions. Crucially, reading times show an agreement attraction pattern with the slowdown associated with a subject–verb number agreement violation being much reduced in the presence of a plural attractor (interaction between grammaticality and attractor number: *t* = −3.18).

**TABLE 5:** Results of the linear mixed effects model for trials on which the head-matching adjective was chosen (using log transformed RTs).

**Parameter**	**Estimate**	**Std. error**	***t*-value**
**Verb region**			
Intercept	5.853	0.043	137.87
Grammaticality	0.002	0.005	0.35
Attractor number	−0.002	0.005	−0.28
Grammaticality × attractor number	−0.004	0.005	−0.85
**Spillover region**			
Intercept	5.894	0.043	136.18
Grammaticality	−0.036	0.005	−6.77
Attractor number	0.015	0.005	2.78
Grammaticality × attractor number	−0.017	0.005	−3.18

**FIGURE 6:**
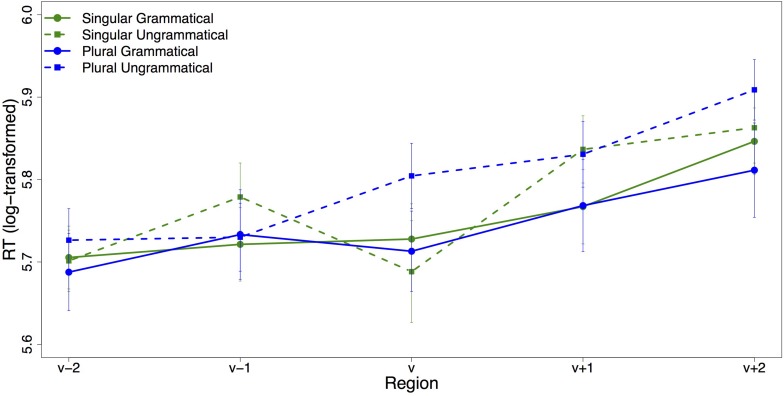
Region-by-region mean reading times for trials on which the attractor-matching adjective was chosen. Error bars indicate standard error of the mean.

Average log-transformed reading times for trials on which participants chose the attractor-matching adjective are plotted in Figure 6. It should be noted that the high accuracy on the adjective choice task meant that the sample size for this analysis was extremely small, so we do not present a statistical analysis. Visual inspection of the plot reveals a very different pattern than for the head noun compatible adjective response trials with a slowdown for ungrammatical sentences with a plural attractor in the verb region. However, this data is suggestive at best and we refrain from interpreting it.

## Discussion

As expected, participants showed a clear agreement attraction effect in the overall self-paced reading data. If misretrieval of the attractor triggers structural reanalysis, this should be reflected in participants’ choosing the attractor-matching adjective. In fact, we did find that participants chose the attractor-matching adjective more frequently in the agreement attraction configuration. However, the subset of trials on which this happened was small across all conditions. If we take the speeded-acceptability data from the attraction norming study as a very rough proxy of how frequently participants experienced attraction in these materials, we can compare this to the rate of misinterpretation in the adjective-choice task. In the norming study, the rate of accepting ungrammatical sentences with a plural attractor was 16.9% higher when the attractor was plural (25.1% acceptance rate) compared to when it was singular (8.2% acceptance rate). In contrast, the rate of choosing the attractor-matching adjective was only 3.3% higher in ungrammatical sentences when the attractor was plural (16.6%) compared to ungrammatical sentences in which the attractor was singular (13.3%). While we acknowledge that this is a very rough estimate, we do think it suggests that misretrieval of the attractor during agreement processing frequently occurs without resulting in the misinterpretation of the attractor as the subject.

Further evidence against the idea that agreement attraction generally results in reanalysis comes from the response contingent analysis of the self-paced reading data. There is a clear pattern of agreement attraction in the trials on which the correct head-matching adjective was chosen (the majority of trials). Under a view in which misretrieval of the attractor leads the parser to reanalyze it as the subject, we would expect less attraction on these trials than in the overall data since misretrieval should result in choosing the attractor-matching adjective. Unfortunately, the subset of trials on which the attractor-matching adjective was chosen is too small for statistical analysis and we cannot easily compare the rate of attraction in the self-paced reading data based on adjective choice.

Although the results demonstrate that error-driven retrieval for agreement checking is not inextricably linked to reanalysis, they also suggest that misretrieval and misinterpretation are not completely independent. The advantage of the dual-task paradigm is that it provides an explicit measure of what participants interpreted as the subject on each individual trial: while comprehenders very rarely chose the adjective compatible with the attractor, they did so significantly more frequently in ungrammatical sentences with plural attractors. This suggests that the attractor is at least occasionally misrepresented as the subject and that error-driven retrieval in response to the detection of an agreement violation might contribute to the likelihood of structural reanalysis.

The nature of the task meant that the number marking always had to appear on copular ‘*be*,’ which is semantically impoverished, but it is possible that misretrieval of the attractor triggers restructuring if the verb simultaneously contains additional semantic cues in favor of the alternative structure [see Cunnings and Sturt (2018) for data suggesting implausible verb-object combinations are susceptible to semantic facilitative similarity-based interference]. Moreover, the type of materials could have made reanalysis less likely: the subject’s head noun was always the first noun in the sentence, making it very salient. In fact, participants could have used a task-specific strategy in which they rely on sentence-initial position to establish subjecthood in the adjective choice task. In future research, this potential task-specific heuristic could be prevented by including items in which subjecthood and sentence-initial position are dissociated.

While the results of the present study point toward an interaction between error-driven retrieval for agreement checking and misinterpretation, it should be acknowledged that a potential explanation for this pattern can be provided without assuming that it is directly linked to agreement attraction as such. The average reading times for trials with an attractor-matching response were faster than for trials on which the head-matching adjective was chosen. Again, it needs to be noted that this was only a very small subset of trials. Nevertheless, this would be compatible with a situation in which attractor-matching responses might occur on trials on which participants were not paying attention. In that case, the mental representation of the subject might be less well encoded and less stable than usual. On some of these trials, the attractor might even have been analyzed as the subject before the verb was encountered. Without a robust structural representation of the input prior to the verb, it is possible that in these cases neither of the NPs is in subject position when the verb is encountered. The plural marking on the verb could then have served as a cue to pick the NP with the matching number feature as the subject, explaining why attractor-matching adjectives were chosen more frequently in ungrammatical sentences with plural attractors. Although this relies on a match between the attractor’s number feature and the retrieval cues of the verb, it is not identical to the mechanism we usually assume for agreement attraction. Unfortunately, we have no data on how confident participants were about their adjective choices. If attractor compatible adjective choices really were due to inattention, participants would be expected to be less confident about their choice on these trials.

Overall, the results of this study indicate that error-driven retrieval triggered by the detection of a subject–verb agreement violation only sometimes results in the misinterpretation of the attractor as the subject. This suggests that attraction effects in comprehension might reflect two different processes: In some cases, misretrieval of the attractor triggers structural reanalysis and results in the misinterpretation of the attractor as the subject. However, agreement attraction seems to often index a low-level feature checking operation in the following sense: Comprehenders predict the number marking of the verb based on the subject and retrieve the agreement controller if the verb does not match this prediction to check whether its number feature can license the number marking on the verb. If, it is no longer perceived as an agreement violation. This relies on a low-level morphosyntactic checking mechanisms in which only the retrieved item’s number feature is checked, since the misretrieved attractor does not match all of the verb’s retrieval cues.

A reviewer notes that one possible alternative explanation of these data is that misinterpretation does occur in tandem with agreement attraction, but that participants ‘fix’ the misinterpretation at a later stage process when the adjective is encountered. In other words, participants could have initially integrated the adjective with the misinterpretation driven by the agreement configuration, but then re-checked the interpretation by retrieving the initial noun in the sentence, such that this reanalysis would yield the correct interpretation. Although we don’t have any evidence for this two-stage strategy in the current data, we agree that it will be important for future work to more carefully evaluate this possibility with a more time-sensitive interpretation measure.

### The Final Representation of Agreement Attraction Sentences

The question whether the misretrieval of the attractor in agreement processing triggers reanalysis has important implications for whether grammatical illusions can arise with mental representations that are not actually grammatical. If misretrieval of the attractor necessarily triggers restructuring, agreement attraction would only occur when the verb’s number marking is actually licensed by the final representation: with the plural attractor misrepresented in subject position, there would be no agreement violation. This would suggest that grammatical illusions arise on the basis of final representations that are not consistent with the input, but are consistent with the grammar.

In contrast, if the output of retrieval is only used to check that the number marking on the verb is consistent with the number feature of the agreement controller, misretrieval of a number matching attractor would simply signal that there is no agreement violation after all. However, the final structural representation in memory would still contain a number mismatch between the actual subject and the verb and would therefore be consistent with the input but not the grammar.

If a number matching attractor is retrieved instead of the number-mismatching subject, that signals that there is no agreement violation after all. Due to this illusory licensing of the verb’s number marking by the attractor, the comprehender does not perceive the sentence to be ungrammatical. Consequently, there is no additional repair process to revise the subject’s or the verb’s number and the final representation remains inconsistent with the grammar. That might be considered a problem for a low-level feature checking account if we assume a framework in which interpretations have to be derived from structural representations consistent with the grammar. However, it very much depends on when exactly we think agreement has to be licensed in online processing. If the verb’s number only matters at the point at which it is integrated into the structure, illusory checking due to misretrieval of the number-matching attractor would be entirely sufficient and the discrepancy between the structure and the features that were checked does not matter.

The results of our study suggests an account of agreement attraction that does not necessarily involve reanalysis. This means that the illusory licensing of an agreement violation must be possible without a final mental representation of the sentence in which it is actually licensed. However, the slightly higher proportion of attractor-matching adjective choices in agreement attraction configurations suggests that a subset of trials on which the attractor is misretrieved does lead to the misrepresentation of the attractor as the subject. In this subset, the final mental representation does actually license the verb’s number marking. This suggests that what we observe as the phenomenon of agreement attraction in measures such as speeded acceptability judgments and self-paced reading may not reflect exactly the same underlying process on all trials.

### A Third Possibility: Revising the Subject’s Number Feature

The results of the present study suggest that the error-driven retrieval process that results in agreement attraction is a low-level rechecking process that does not usually have any structural impact. However, one could imagine a third possibility that falls in between a structural reanalysis account and a simple feature-checking model. It is possible that the representation of the sentence is altered based on the retrieval output, but without structural reanalysis. In particular, the parser could use the number feature of the erroneously retrieved attractor to substitute the number feature of the subject as it was originally encoded in memory. For example, in a sentence with an agreement violation and a number-matching attractor, such as ‘*The key to the cabinets are rusty*,’ the process would be the following: The subject is correctly encoded as singular and the parser predicts a singular verb. Upon encountering ‘*are*,’ there is a mismatch between the number feature of the prediction and the bottom-up input, which triggers a search for the agreement controller in memory. If the number-matching attractor is erroneously retrieved, it’s number feature is used to “correct” the subject’s current number feature. Unlike the pure rechecking process, this account predicts interpretive consequences of misretrieval, but would result in a final representation that is consistent with the grammar as a whole and does not contain an agreement violation.

If misretrieval of the number matching attractor results in the change of the subject’s number feature, this could in a sense be considered a representational account since it involves misrepresenting the number of the subject. However, it would be fundamentally different from other misrepresentation accounts: In representational accounts like feature percolation (Bock and Eberhard, 1993; Vigliocco et al., 1995; Eberhard, 1997; Franck et al., 2002) and the Marking and Morphing model (Bock et al., 2001; Eberhard et al., 2005), agreement attraction is a consequence of misencoding the subject’s number feature prior to encountering the verb. In contrast, if the parser changes the subject’s number feature based on the output of retrieval in agreement processing, misrepresenting the subject’s number information would be a consequence of agreement attraction, rather than the cause of it. The main argument against representational accounts of agreement attraction in comprehension is the grammatical asymmetry (Wagers et al., 2009): If the subject’s number is misrepresented in the presence of a plural attractor, we would expect grammatical sentences to sometimes be perceived as containing an agreement violation. This illusion of ungrammaticality has generally not been found in the literature (Wagers et al., 2009; but cf. Hammerly et al., 2018). However, if misrepresentation of the subject’s number feature occurs not before the verb is encountered but as a consequence of encountering a plural verb, this would account for the lack of an illusion of ungrammaticality.

As discussed in the section on agreement and interpretation, there is some data that suggest that comprehenders do misinterpret the subject as plural in agreement attraction configurations (Patson and Husband, 2016; Brehm et al., 2019). However, this was measured by non-literal plural responses to comprehension questions, which were also higher when the local noun was singular and only the verb was plural. This is not predicted by representational accounts of agreement attraction and is more consistent with a noisy-channel model of comprehension, or an account in which the answers to explicit comprehension questions do not necessarily show an accurate reflection of the representation built during the earlier processing of the sentence. Although the data suggest that agreement attraction does not arise as the consequence of number misrepresentation, they do not speak to the question whether number misrepresentation might arise as a result of misretrieving the attractor.

Consequently, while the results from Patson and Husband (2016) and Brehm et al. (2019) are intriguing, they do not provide conclusive evidence that agreement attraction arises from comprehenders misrepresenting the subject’s number feature due to the presence of a plural attractor. Nevertheless, in light of the recent evidence that comprehenders sometimes carry out structural repairs on anomalous input, the possibility that comprehenders end up misrepresenting the subject’s number information in agreement attraction cannot be dismissed without further research.

## Conclusion

We explored the relationship between the output of error-driven retrieval in agreement processing and the final structural representation of the sentence. We used a novel dual-task design to assess whether comprehenders misinterpret the attractor as the subject when they experience agreement attraction. The results suggest that comprehenders do not misinterpret the attractor as the subject on all trials on which agreement attraction occurs, indicating that misretrieval of the attractor does not necessarily trigger restructuring. While this implies that subject–verb agreement attraction is not a straightforward reflection of reanalysis, misretrieval of the attractor does appear to increase the likelihood of misinterpreting the attractor as the subject. This suggests that the error-driven retrieval process in agreement checking often involves low-level feature checking without integrating the output of retrieval into the agreement controller’s position in the mental representation. Nevertheless, in a subset of cases, this low-level feature checking does serve as an impetus for structural reanalysis.

Since the data suggest that structural reanalysis is not necessarily triggered when the attractor is misretrieved, this indicates that illusory licensing can occur even if there is no actual licensing in the final mental representation. Whether this discrepancy will hold for other grammatical illusions is unclear; agreement as such does not contribute to the interpretation of a sentence and, unlike grammatical illusions involving dependencies that cannot be predicted such as reflexives or VP-ellipsis, it is an error-driven phenomenon. This potential difference between agreement attraction and non-error driven grammatical illusions certainly warrants further investigation.

## Ethics Statement

The protocol was approved by the University of Maryland Institutional Review Board. All subjects gave written informed consent.

## Author Contributions

ZS initiated the project and designed the experiments in collaboration with EL and DP. ZS ran the study and performed the analysis under the supervision of EL. ZS wrote the first draft of the manuscript. All authors critically revised the drafts.

## Conflict of Interest Statement

The authors declare that the research was conducted in the absence of any commercial or financial relationships that could be construed as a potential conflict of interest.

## Footnotes

1 The model also converged with by-subject and by-item random intercepts and by-subject random slopes for attractor number, but the significance of the effects does not depend on which of these models is used.

2 This led to the exclusion of 3 out of 6057 observations in the regions of analysis.
