# Supplementary material for: Error-Driven Retrieval in Agreement Attraction Rarely Leads to Misinterpretation
Source: Front Psychol. 2019 May 7;10:1002. doi: 10.3389/fpsyg.2019.01002 (PMC6524724; doi:10.3389/fpsyg.2019.01002)
Supplement: Supplementary file 1 [file Table_1.DOCX]

Supplementary Material

# Experimental items

# 1. The woman by the window was most definitely amused / dusty

# 2. The flower by the bed was most definitely blooming / made

# 3. The notebook for the class was unfortunately rather ripped / difficult

# 4. The laptop on the magazine was most certainly overheating / wordy

# 5. The jack-o'-lantern near the child was certainly rather carved / upset

# 6. The baby near the pillow was quite noticeably happy / fluffy

# 7. The woman with the shopping bag was nearly always sad / ripping

# 8. The cloud above the car was obviously extraordinarily fluffy / flashy

# 9. The bottle with the logo was very remarkably leaky / clever

# 10. The boy by the tree is really very chubby / green

# 11. The bed by the lamp was undoubtedly quite comfortable / bright

# 12. The student with the textbook was nearly always cheerful / read

# 13. The pool behind the gate was almost always full / stuck

# 14. The band near the van was probably pretty rested / fuel-efficient

# 15. The cat under the chair was most likely sleepy / wobbly

# 16. The girl in the movie was rather unusually tall / long

# 17. The author of the paper was truly remarkably outraged / well-written

# 18. The novel next to the interviewer was entirely too lengthy / loud

# 19. The glass behind the curtain was most definitely shattered / tattered

# 20. The vacation for the family was still profoundly satisfying / jet-lagged

# 21. The corn from the field was actually very tasty / fruitful

# 22. The cake for the woman was certainly intentionally decorated / kind

# 23. The painting of the dog was surprisingly very artistic / tired

# 24. The coffee for the office was usually rather burnt / quiet

# 25. The football near the tree was generally fairly flat / tall

# 26. The plant next to the gnome was really quite leafy / scary

# 27. The apple near the cabinet was luckily fairly fresh / childproof

# 28. The key for the door was apparently quite lost / open

# 29. The couple with the stroller was seemingly rather tired / damaged

# 30. The movie with the dinosaur was really quite well-made / extinct

# 31. The man with the crutch was unfortunately very unhealthy / bent

# 32. The music for the concert was clearly very difficult / crowded

# 33. The eraser beside the pencil was evidently rather round / sharp

# 34. The owner of the store was rarely ever friendly / cluttered

# 35. The person by the lounge was rarely ever excited / vacant

# 36. The house by the waterfall was probably extremely expensive / turbulent

# 37. The backpack with the zipper was unfortunately extremely heavy / broken

# 38. The book near the window was only slightly tattered / transparent

# 39. The note for the proprietor was surprisingly very illegible / happy

# 40. The umbrella near the closet was certainly very wet / empty

# 41. The towel on the shelf was almost entirely shredded / tidy

# 42. The photograph for the newspaper was certainly well cropped / proofread

# 43. The child with the basketball was usually fairly happy / inflated

# 44. The water in the cooler was always remarkably refreshing / damaged

# 45. The runner in the marathon was certainly very athletic / long

# 46. The ground under the water fountain was occasionally somewhat slippery / loud

# 47. The elf with the present was almost always joyful / wrapped

# 48. The food for the event was unquestionably very delicious / organized
